# Supplementary material for: Patterns of Intron Gain and Loss in Fungi
Source: PLoS Biol. 2004 Nov 30;2(12):e422. doi: 10.1371/journal.pbio.0020422 (PMC532390; doi:10.1371/journal.pbio.0020422)
Supplement: Table S1 — Also available at http://genes.mit.edu/NielsenEtAl/. (4.3 MB ZIP). [file pbio.0020422.st001.zip › NielsenEtAl/html/1253.html]

AN7542.1.NCU07379.1.MG00342.1.FG01313.1


```
 CLUSTAL W (1.82) Multiple Sequence Alignments - Introns Inserted


Sequence 1: NCU07379.1	365 aa
Sequence 2: MG00342.1	426 aa
Sequence 3: FG01313.1	361 aa
Sequence 4: AN7542.1	446 aa
Alignment Length: 485 aa
Number Identitical Residues: 84 aa
Alignment Score (without introns) 4440


MG00342.1 	MLS1CHLGGGGGFSTRTRPYVLFISPYQKLIISVV2GVSSPPMQSYEGSG~GSDGGHDKS
NCU07379.1	---~----------------------------MTS~SLPLQPLATYAEDP~HS--GPDSN
FG01313.1 	---~-------------------MSVFNN---HLA~GATERSFSSSLSPD~RIIASSEDD
AN7542.1  	---~--------------MTSISSRPIPLDLNAMD~SLNRQSLAQSASPA1GQDTPGMRG
          	                                    .    .:                .

MG00342.1 	PLSSIPSNFLRN-LTDKRTKS1D~APAPKRRGPKPDSKPALTRRQELNRQAQR2THRERK
NCU07379.1	PLSTLNLTILKS-LSDKKTR-~D1GQTPKRRGPKPDSKPALTRRQELNRQAQR2THRERK
FG01313.1 	NAAALDPEIYKS-LADKRTTR~D1GNPPKKRGPKPNSKPALTRRQELNRQAQR2THRERK
AN7542.1  	SISTDFFKFFGAGGQAKKTTR~D1GQPAKRRGPKPDSKPALTRRQELNRQAQR2THRERK
          	  ::    :   .   *:*   * . ..*:*****:***************** ******

MG00342.1 	ELYIKALEDEVLRLKETYSNVSQAK-----------DNLSNENRQLKQLLAQNGISFGGA
NCU07379.1	ELYIKALEDEVLRLKEIFGTISQDK-----------ERLAEENRRLKAKLAQN-DAAGDP
FG01313.1 	ELYIKALEDEVLRLKEVFSNVSLDR-----------ERLADENKRLRDTLVQAGLQHSSP
AN7542.1  	EQYIRALESEVARLREAYTQEISAANLAVHQHREMANSISEENNMLKEILVAHNINYDAE
          	* **:***.** **:* :       . :  .  . :: :::**. *:  *.  .   .  

MG00342.1 	MGIASGMMDDGS-IDYASSSATGSGYGGGPASSHTSAFTPSPLASDTANSQQYSRTPTGH
NCU07379.1	IAADTNFFDQ----MVSSPSGPSPGYTSSSSMLGSESYLPGGPSSQTTFPSPPSSTTTSP
FG01313.1 	HVAG---------------SATSRGFDSNNTGS-------SPLTSQSTAPSVGSPMHLPP
AN7542.1  	LERRRAERQMGSGFHSSPVGAGSVGSQTGIASLTAHTYSTPPTTVSSGMSPKFNGVEKFD
          	        . .:.   :. .. . *   . :   : :  .   : .:  .   .      

MG00342.1 	GSLSPHPGGHHQHTNAQHHPRHGAQPLNNPGV------DYEQAGIDFVLA2LEKPCMDHL
NCU07379.1	-AYRPPLGGPRDFNTVLGDGAGVMVSKRDPDL------DYDQAGIDFVLT2LERPCMEHM
FG01313.1 	AVNQGFVSGQQQHG--------VPRPLYHGNP------ELEQAGIDFVLT2LERPCMTHI
AN7542.1  	FSSVPDVGPNNQGIATVPCDALAAIDRRPPIQGAGIFEDNPQLQIDFILA2LESPCRDHT
          	       .  .:                    .:.  .:  *  ***:*: ** **  * 

MG00342.1 	PWLLERGTETGG----EACGHALMASCPPEPFSELTPDIPFGYSNVKNGELD--------
NCU07379.1	TWLLERGTGTEDRAQREPCGHALMASCPPEPFSELSPESPFGHTNTIHAHAHGHPPMPTS
FG01313.1 	QFMVERASEPEG----EPCGHALMASCPPDPSPESRPGLPFGKIHIPVDGEP--------
AN7542.1  	DYLCRRSITEADDEDMPFSGHALMATCPPPSYIANTTSEQT-------------------
          	 :: .*.    .  .   .******:*** .     .                       

MG00342.1 	---SGQRTWELSKGDLNTLLDLSKRLNLDGEITPVMAWGMVLAHPRLGEMTPKDFKKLTE
NCU07379.1	ISGGGQRTWELSKADLATLLDLSKRLDLDGEITPVMAWGMILAHPRLAELQLEDFARLTE
FG01313.1 	----SQKTWEVPKADLATLLDLSKSIDLDGEVTPIMSWGILMSHPRANELEAADFRKLSE
AN7542.1  	---YPHKTYDLPHANLTTLLNLSRQLVTEGQVTPIMALQALKNHEMYTKLTRDDVKLIMD
          	     ::*:::.:.:* ***:**: :  :*::**:*:   :  *    ::   *.  : :

MG00342.1 	ELCGK~VR--------------CYG2FGAVMEEFEARDAIENVFSTKPDSFHQSYSTEYG
NCU07379.1	ELGSK~VR--------------CYG~----------------------------------
FG01313.1 	DLVRK~VR--------------CYG2FGAVMEEFEVRDAIDNVFSGKDGMMYE-------
AN7542.1  	TLENK0INRTSSQDSINGLERLCLL~ILPAPVKDLSGRALRQFFLQLTPYRAELSYRLCG
          	 *  * :. :::..: .. .  *     .   .     :  .          .      .

MG00342.1 	GGR--------~--~-
NCU07379.1	-----------~--~-
FG01313.1 	-----------~--~-
AN7542.1  	CDRLTVFNCAG0IG0F
          	 .  :  ..:.  .
```
